# What Is the Diagnosis in Patients with Type 2 Diabetes Who Have a Painful Shoulder? Results from a Prospective Cross-Sectional Study

**DOI:** 10.3390/jcm9124097

**Published:** 2020-12-18

**Authors:** Login Ahmed S. Alabdali, Jasmien Jaeken, Nens van Alfen, Geert-Jan Dinant, Rob A. P. Borghans, Ramon P. G. Ottenheijm

**Affiliations:** 1 Department of Family Medicine, CAPHRI Care and Public Health Research Institute, Maastricht University, P.O. Box 616, 6200 MD Maastricht, The Netherlands; geertjan.dinant@maastrichtuniversity.nl (G.-J.D.); ramon.ottenheijm@maastrichtuniversity.nl (R.P.G.O.); 2 Ministry of Education, Riyadh 12435, Saudi Arabia; 3 Department of Public Health and Primary Care, Catholic University of Leuven, Kapucijnenvoer 33, B-3000 Leuven, Belgium; j.jaeken@alumni.maastrichtuniversity.nl; 4 Department of Neurology and Clinical Neurophysiology, Radboud University Medical Center, Donders Institute for Brain Cognition and Behaviour, 6500 HB Nijmegen, The Netherlands; Nens.vanAlfen@radboudumc.nl; 5 Department of Radiology, Zuyderland Medical Centre, 6162 BG Sittard-Geleen, The Netherlands; R.borghans@zuyderland.nl

**Keywords:** type 2 diabetes, physical examination, diagnosis, ultrasound, shoulder pain, adhesive capsulitis, subacromial pain syndrome

## Abstract

Background: Patients with diabetes mellitus have higher risk of developing shoulder pathology. However, only adhesive capsulitis is addressed in shoulder pain guidelines as a disorder associated with diabetes. Yet, patients with diabetes are at risk of having several other shoulder disorders, including focal neuropathy. Our aim was to quantify the presence of shoulder disorders using physical examination and ultrasound imaging in patients with type 2 diabetes (T2DM) suffering from shoulder pain in general practice. Methods: In this prospective cross-sectional study, patients with T2DM who had had a painful shoulder for at least four weeks were included. Patients filled out a questionnaire and underwent a physical examination of the shoulders and feet and ultrasound imaging of the shoulder. Results: A total of 66 patients were included, of whom 40.9% (*n* = 27) had bilateral complaints resulting in 93 symptomatic shoulders. Subacromial pain syndrome was most frequently diagnosed by physical examination (66.6%, 95% CI 51.6–72.0%; *p* < 0.0001), while ultrasound imaging showed that subacromial disorders were statistically significantly the most prevalent (90.3%, 95% CI 81.9–95.2%). Only two patients (3%) were diagnosed with neuropathic shoulder pain. Conclusion: When choosing treatment, general practitioners should be aware that in patients with T2DM the subacromial region is most frequently affected.

## 1. Introduction

Patients with type 2 diabetes mellitus (T2DM) are at increased risk for shoulder disorders, such as adhesive capsulitis and rotator cuff disorders (e.g., tears). The prevalence of adhesive capsulitis ranges from 4 to 30% in patients with diabetes, compared to up to 10% in patients without diabetes [1,2,3,4], while rotator cuff tears are also more frequently observed in patients with diabetes [5,6,7,8]. Lesser known is that adhesive capsulitis is also associated with diabetic neuropathy [9].

The exact pathophysiology of the increased risk of shoulder disorders in patients with diabetes remains uncertain, but there is evidence that the shoulder can be affected through two pathophysiological pathways: connective tissue damage to the rotator cuff tendons and joint capsule, and peripheral or autonomic neuropathy. Connective tissue damage seems to be caused by abnormal collagen disposition in the periarticular connective tissues, due to the formation of advanced glycosylation end-products. This alters the structural matrix and the mechanical properties of these tissues. Additionally, it is hypothesized that the altered glucose metabolism in patients with diabetes causes functional as well as structural changes to the peripheral nerve system, which ultimately leads to neuropathy [5,10]. About 50% of patients with diabetes mellitus develop diabetic neuropathy [11,12], which can be classified as generalized symmetrical polyneuropathy or (multi)focal asymmetrical neuropathy [11,13]. If patients with subclinical levels of neuropathy are included, the prevalence might exceed 90% [14,15]. However, in patients with diabetes, shoulder involvement has been described in case studies, but is currently regarded as rare [16,17,18].

While rotator cuff disorders (also named subacromial pain syndrome, abbreviated to SAPS [19,20]) are widely considered the most common cause of shoulder pain in general practice [21,22,23], diabetes mellitus is currently only associated with adhesive capsulitis in shoulder pain guidelines, while neuropathy is not addressed at all [20,24,25]. However, to ensure optimal treatment outcomes, it is important to identify the underlying disorder in patients with diabetes to prevent the development of chronic shoulder pain. For example, patients with SAPS and neuropathic pain might benefit more from treatment targeted to the neuropathic pain [26]. This seems important especially in patients with T2DM, as shoulder pain might negatively influence physical activity, which is considered to be the cornerstone of diabetes treatment. Therefore, inadequate treatment of shoulder pain might negatively influence diabetes treatment, eventually leading to a vicious circle [27,28].

To our knowledge, no studies have been conducted to establish the frequency of underlying shoulder disorders in patients with T2DM suffering from shoulder pain. In order to prevent the development of chronic shoulder pain in patients with diabetes, more insight is needed into which specific types of shoulder pathology are prevalent in T2DM. Given the complex nature of shoulder pain, the primary aim of this study was to quantify the presence of specific shoulder disorders using physical examination and musculoskeletal ultrasound imaging in patients with type 2 diabetes mellitus (T2DM) in general practice. Secondarily, we sought to examine the relationship of the specific disorders with the presence of a diabetic neuropathy. With this information, we can increase general practitioners (GP) awareness of this specific problem in T2DM.

## 2. Methods

### 2.1. Participants and Study Design

This was a prospective cross-sectional study of patients with T2DM suffering from shoulder pain in general practice. Patients were invited to this study while participating in a questionnaire study assessing the prevalence of upper extremity musculoskeletal disorders (manuscript under review). During their annual check-ups in general practice, they had been asked to participate in a questionnaire study, which also included an invitation for the current study that was only intended for patients who had shoulder pain. All eligible participants from the questionnaire study were invited to participate. To increase recruitment, a flyer announcing this study was sent out to physiotherapy and general practices in the Sittard-Geleen area in the Netherlands. During our inclusion period, for which we had a time frame of nine months (March 2018 to November 2018), we included all eligible patients. Inclusion criteria were being aged between 30 and 70 years, and having shoulder pain that had lasted longer than 4 weeks. Exclusion criteria were inability to complete the assessments, and inability to give informed consent. After providing informed consent, patients underwent the following assessments: a questionnaire addressing shoulder pain including co-morbidity and diabetes-related questions, standardized physical examination of the shoulder, neurological examination of the feet, and ultrasound imaging of the shoulder (see below). All examinations were carried out in the Meditta Medical Center in Echt, a diagnostic center, between March 2018 and November 2018. This study was approved by the Medical Ethics Committee of Zuyderland Medical Centre (METC-Z 17-N-165).

### 2.2. Questionnaire

The following demographic and shoulder-specific information was collected: an 11-point Numerical Rating Scale, affected side and dominant arm, pain onset (sudden or gradual), neck pain involvement, and if there was a history of rheumatoid arthritis, osteoarthritis, and any other joint inflammation. Additionally, we collected information about the most recent HBA1c value, the current body mass index (BMI), and year of their diabetes diagnosis.

### 2.3. Ultrasound Imaging

Ultrasound examination of the shoulder was the first examination participants underwent, and was performed by one of the three participating, experienced radiologists, using a Phillips EPIQ 7G (Philips Healthcare, Eindhoven, the Netherlands) with a high-resolution, multi-frequency 4–18 MHz linear transducer. A standardized protocol was performed following the technical guidelines of the European Society of Musculoskeletal Radiology for shoulder scanning [29]. In patients with diabetes, both shoulders were assessed. The following structures were assessed: the tendon of the supraspinatus, infraspinatus, subscapularis, and the long head of biceps, as well as the subacromial-subdeltoid bursa, rotator interval, glenohumeral recess, and acromioclavicular joint (AC joint). We used standardized criteria for subacromial pathology previously defined by us, and added criteria for adhesive capsulitis (see Supplement Table S1) [30,31,32]. The following disorders were recorded: dislocation of the long head of biceps tendon, biceps tenosynovitis, (calcific) tendinopathy, any kind of tendon tear, bursitis, adhesive capsulitis, paralabral cyst, and acromioclavicular osteoarthritis. Next, they were categorized by their anatomical location as subacromial, glenohumeral, or other disorders, by following a detailed standard operating procedure (see Supplement Table S1 for full details).

### 2.4. Physical Examination

One trained medical doctor of our study team (LA), blinded to the ultrasound imaging results, carried out a structured physical examination of both shoulders, hands, and a neurological examination of the feet.

**Figure 1:**
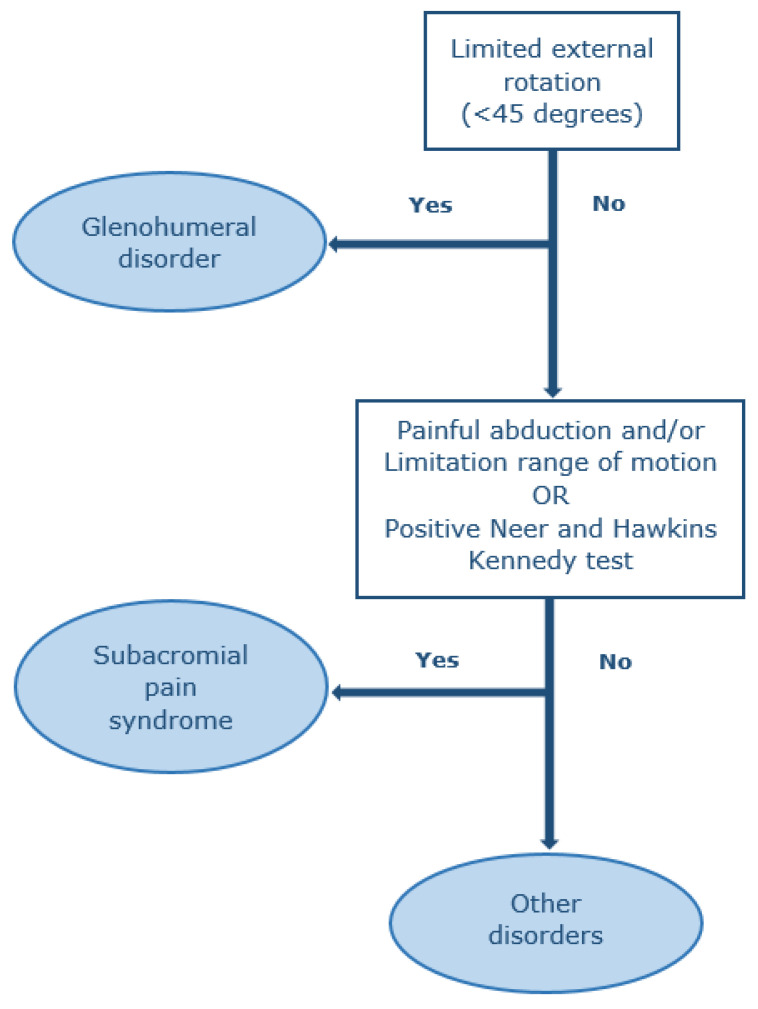
Diagnosis based on physical examination by a mutually excluding method.

Shoulders were examined according to the shoulder pain guidelines of the Dutch College of General Practitioners prevailing at the time of conducting the study [33]. One year later (2019), these guidelines were revised. Therefore, we used the revised version for subsequent classification of the shoulder disorders [20]. Shoulders were inspected for muscle wasting and scapular winging. Active and passive abduction and passive external rotation were carried out to assess for pain and range of motion was visually estimated. Additionally, the Hawkins–Kennedy test and the Neer impingement test were carried out [34,35]. The 10-item Douleur Neuropathique questionnaire was performed to assess neuropathic shoulder pain; a commonly used questionnaire including physical examination for screening and diagnosing neuropathic pain in patients with neurological complaints, valid for the Dutch population, and validated in diabetes patients [36,37,38]. In the Dutch population, the cut-off score for neuropathic pain is considered ≥5 (out of 10 points) [39]. Physical examination findings were used to diagnose the following disorders: SAPS, a glenohumeral or an “other disorder”, and categorized using a mutually exclusive method leading to a single diagnosis (Figure 1) [20]. Glenohumeral disorders were defined by an external rotation range of motion of less than 45 degrees, while SAPS was defined by either a painful abduction (including a painful arc) with or without a limited range of motion during abduction, or a positive Hawkins–Kennedy and Neer test. An “other disorder” was defined as not having any of the two previous disorders.

Examination of the feet consisted of two parts: an interview part to evaluate neuropathic symptoms, and a neurological examination to evaluate signs. Participants were asked if they had a burning, tingling, or numbness sensation, and whether they felt like they were walking on cotton wool. The neurological examination included inspection for distal muscle weakness or atrophy, ankle reflexes, touch sensation, and vibration sense. The diagnosis of clinical polyneuropathy was based on a combination of symptoms and signs: presence of at least one symptom in combination with decreased or absent ankle reflexes, and decreased or absent distal sensation (vibration sense and/or touch sensation) with or without distal muscle weakness. For subclinical polyneuropathy, the abovementioned signs had to be present in the absence of symptoms. All examinations including criteria for pathology are presented in the Supplement (see Supplement Table S2) [40,41,42,43,44,45,46]. Finally, the hands were examined for the presence of a positive tabletop sign or prayer sign as a manifestation of stiff hand syndrome (see Supplement Table S3) [47,48,49].

### 2.5. Statistical Analysis

Continuous variables were tested for normal distribution. Descriptive statistics are presented for continuous variables as means with standard deviations, and categorical variables data are presented as absolute frequencies and percentages. Statistical differences for physical-examination-diagnosed shoulder disorders were calculated using the Chi-square test to compare between the three diagnostic groups. All statistical analysis were performed using IBM SPSS Statistics for Windows (version 25.0, Armonk, NY, USA).

## 3. Results

Table 1 presents the demographic and shoulder pain characteristics, as well as the results of the neurological and hand examination. A total of 66 patients with T2DM suffering from shoulder pain could be included during the study period. In total, 39 were recruited via the questionnaire study, and 27 via the flyers. The mean age was 63.0 years and 28.8% (*n* = 19) was female. Bilateral complaints were present in 41% of the patients (*n* = 27), resulting in 93 symptomatic shoulders and 39 asymptomatic shoulders.

### 3.1. Physical-Examination-Diagnosed Symptomatic Shoulder Disorders

The results of the shoulder diagnosis based on physical examination are presented in Table 1. SAPS was statistically the most frequently observed disorder in symptomatic shoulders (66.6%, 95% CI 51.6–72.0%), followed by glenohumeral disorders (18.2%, 95% CI 11.3–27.9%; *p* < 0.0001 versus SAPS), and then “other disorder” (16.1%, 95% CI 12.1–29.1%; *p* < 0.0001 versus SAPS). Neuropathic shoulder pain was observed in two patients (3.2%); one patient was diagnosed with SAPS, the other with an “other disorder”.

### 3.2. Polyneuropathy

Clinical polyneuropathy of the feet was observed in 28.8% (*n* = 19), while subclinical polyneuropathy was present in 56.1% (*n* = 37). In patients with a glenohumeral disorder, polyneuropathy was most commonly observed (29.4%, *n* = 5).

### 3.3. Ultrasound-Diagnosed Shoulder Disorders

Table 2 presents the results of the ultrasound findings in both symptomatic and asymptomatic shoulders. Subacromial disorders were most frequently diagnosed in both symptomatic and asymptomatic shoulders (90.3% (*n* = 84) and 76.9% (*n* = 30), respectively) with rotator cuff disorders being most prevalent. This was followed by osteoarthritis of the AC joint (59.1% (*n* = 55) and 43.5% (*n* = 17), respectively) and then glenohumeral disorders (8.6%, *n* = 8) in symptomatic shoulders, while these were not observed in asymptomatic shoulders. Of the rotator cuff disorders, the most frequently observed specific disorder was calcific tendinopathy in both symptomatic and asymptomatic shoulders (80.6% (*n* = 75), 95% CI 70.8–87.8% and 66.7% (*n* = 26), 95%CI 49.7–80.4%, respectively), while rotator cuff tears were least commonly observed (38.7% (*n* = 36), 95% CI 28.9–49.4% and 20.5% (*n* = 8), 95% CI 9.8–36.9%, respectively) (these results are not shown in Table 2). Of the eight patients with a glenohumeral disorder, adhesive capsulitis was present in four cases.

When we matched the results of the physical examination and ultrasound diagnosis, in 97% (56/58) of the patients diagnosed with SAPS, a subacromial disorder was observed by ultrasound imaging. Only two out of 17 patients (12%) diagnosed with a glenohumeral disorder showed ultrasound findings matching this diagnosis.

## 4. Discussion

In this study, using physical examination and ultrasound imaging, we found that the subacromial region is the most frequently affected region in patients with T2DM suffering from shoulder pain. Of these subacromial structures, ultrasound imaging shows that the rotator cuff tendons are most frequently affected, with calcific tendinopathy by far the most common specific disorder. Interestingly, we found that adhesive capsulitis, believed to be frequently diagnosed in patients with T2DM suffering from shoulder pain, was present only in a minority of patients. Moreover, neuropathic shoulder pain can be considered rare as it was present in only two patients.

### 4.1. Diagnosis by Physical Examination and Ultrasound Imaging

Currently, shoulder pain guidelines only associate diabetes with adhesive capsulitis [20,24,25], while we showed that subacromial disorders are by far the most common in this population. In the absence of prevalence studies, a possible explanation for the current misperception might be that adhesive capsulitis is more prevalent among patients with T2DM compared with patients without diabetes [1,2,3,4], but this does not mean that it is the most prevalent shoulder disorder in patients with diabetes.

In line with previous studies conducted in patients with shoulder pain in unselected populations [21,22,23], we also observed that overall, SAPS is the most common cause of shoulder pain in diabetics. The rotator cuff tendons and subacromial-subdeltoid bursa are considered to be the key sources of pathology in this syndrome [50].

SAPS is a generic term, and incorporates all disorders related to subacromial structures including tendinopathy (tendon degeneration), where calcific tendinopathy is seen as a separate diagnosis, tendon tears, and bursitis. From this spectrum of disorders, calcific tendinopathy was the most common specific disorder in our cohort (81%), which echoes the results of previous studies conducted in general practice [51,52,53]. However, in these previous ultrasound studies, the frequency did not exceed 50%, but it is known that diabetes is associated with calcific tendinopathy [54]. Additionally, other rotator cuff tendon disorders were more frequently present in our study. These differences might be explained in pathophysiological terms, and although the exact pathophysiology of tendon disorders in patients with diabetes remains uncertain, there is evidence that abnormal tendon collagen disposition alters the structural matrix and the mechanical properties of the tendons [6]. Through this process, the continuum of tendon pathology might be initiated, where a normal tendon changes into a degenerative tendon (called tendinopathy, different stages are described), and ultimately can tear [55]. Part of this process seems reversible through healing responses, but in patients with diabetes and other endocrine disorders, this healing process can fail; calcium deposits then arise due to a mechanism not yet elucidated [54].

In our study, the large majority of our patients clinically diagnosed with SAPS showed ultrasound findings matching SAPS. In clinical practice, ultrasound findings should always be interpreted together with the clinical findings to determine the cause of symptoms. Several shoulder imaging studies have shown that asymptomatic pathology findings are present, for example supraspinatus tears and osteoarthritis of the AC joint [56,57], and these asymptomatic findings are likely to become symptomatic over time [57]. Surprisingly, we also found ultrasound abnormalities of subacromial pathology in 16 out of 17 patients with a clinical glenohumeral disorder, while only 2 out of these 17 patients had ultrasound evidence of glenohumeral pathology. It might be that ultrasound misses the presence of osteoarthritis of the glenohumeral joint, something we did not assess radiographically in this study. The diagnostic accuracy of ultrasound imaging for osteoarthritis of the glenohumeral joint is unknown, but is likely to be suboptimal.

We also observed that 41% of the patients has bilateral complaints. This is in line with previous studies [58,59], and might be explained in the light of the above and systemic effects of diabetes.

### 4.2. Neuropathy

Although neuropathy is a well-known complication of diabetes, any type of neuropathy of the shoulder is currently regarded as rare [16,17,18]. In line with this observation, we diagnosed only two patients with neuropathic shoulder pain: both these patients were diagnosed with clinical polyneuropathy, but did not have a glenohumeral disorder on physical examination, or adhesive capsulitis on ultrasound imaging. Overall, polyneuropathy on a clinical and subclinical level was present in 39% and 56% of the patients, respectively. This is broadly consistent with a study showing that 38% of patients with T2DM and musculoskeletal disorders had polyneuropathy [9]. We do know that diabetic neuropathy increases the risk of developing adhesive capsulitis of the shoulder, a glenohumeral disorder [9]. In our study, any form of polyneuropathy was most prevalent in patients diagnosed with a glenohumeral disorder.

### 4.3. Strengths and Limitations

Our study had several strengths. We were able to investigate the frequency of the shoulder-diagnosed disorders by two measurements: physical examination and ultrasound imaging. Both indicate that the subacromial region is the most frequently affected structure. The medical doctor who performed the physical examination was blinded to the ultrasound results, which avoided bias for the results of the physical examination.

Our study also had limitations. First, the sample size is small. A larger sample size would have resulted in narrower 95% confidence intervals. Second, we did not include palpation of the AC joint in our protocol, because it was not included in the shoulder pain guidelines of the Dutch College of General Practitioners prevailing at the time of conducting this study. If we had incorporated this in our study, this would also have allowed us to diagnose AC disorders, usually osteoarthritis. In our approach, this disorder is included in the “other disorder” group [33]. It is worth noting that palpation of the AC joint is included in the revised version [20]. Third, neuropathic shoulder pain was diagnosed only by physical examination, while if we had used additional tests, e.g., nerve conducting studies or muscle ultrasound (qualitative or quantitative), it might have increased the number of patients diagnosed with neuropathic shoulder pain. Fourth, the ultrasound diagnostic criteria for glenohumeral disorders such as adhesive capsulitis are debatable as more features are described in the literature, e.g., thickening of the coracohumeral ligament and restriction of external rotation on dynamic scanning [31]. These features are not detected during scanning according to the standardized protocol of the European Society of Musculoskeletal Radiology. For practical purposes, we did not incorporate additional scanning positions necessary to detect these features. This may have introduced an underestimation of the presence of adhesive capsulitis.

### 4.4. Implications for Practice and Future Research

General practitioners and other healthcare professional involved in the care for patients with shoulder pain should be aware that in patients with T2DM suffering from shoulder pain, the subacromial region is the most frequently affected structure. This knowledge can help make a more accurate diagnosis and can influence treatment decisions. When physical examination is not conclusive enough to establish a diagnosis, GPs may consider ultrasound imaging. This seems to be becoming more usual, as GPs increasingly rely on ultrasound imaging [60,61]. Although we showed that polyneuropathy is frequently present, neuropathic shoulder pain seems rare. However, this finding is based only on physical examination results of the shoulder. To assess whether shoulder pain in T2DM might be caused by neuropathy, studies are needed that use techniques to detect neurological denervation, e.g., nerve conducting studies of qualitative or quantitative muscle ultrasound.

## Figures and Tables

**Table 1:** Physical-examination-diagnosed shoulder disorders with the comparison of the demographic and clinical variables in patients with type 2 diabetes mellitus (T2DM), on both patient and shoulder level.

	Total Number of Patients with Shoulder Pain *n* = 66	Symptomatic Shoulders, *n* = 93		
		GH	SAPS	Other Disorder
		*n* = 17 (18.2%, 95% CI: 11.3–27.9) ^#^	*n* = 58 (66.6%, 95% CI: 51.6–72.0) ^#^	*n* = 18 (16.1%, 95% CI: 12.1–29.1) ^#^
Age				
mean ± SD	63.0 ± 6.9	58.8 ± 5.8	61.5 ± 7.0	61.5 ± 7.6
IQR range (years)	38–70	48–70	38–70	52–70
Female sex	19 (28.8)	5 (31.3)	11 (25.0)	3 (42.9)
BMI (kg/m^2^)				
mean ± SD	28.5 ± 4.2	28.1 ± 3.7	28.4 ± 4.5	29.7 ± 3.5
range	16–41.5	23.9–35.9	16–41.5	25.4–34.0
Bilateral shoulder pain	27 (40.9)	8 (50)	15 (34.1)	4 (57.1)
Dominant shoulder affected	26 (39.3)	13 (81.3)	36 (81.8)	7 (100)
NRS				
mean ± SD	5.3 ± 2.1	6.25 ± 1.9	5.2 ± 2.06	4.0 ± 2.6
range	1–9	3–9	1–8	1–8
Pain onset				
sudden	13 (19.7)	2 (11.7)	13 (20.9)	2 ^a^ (11.1)
gradual	51 (79.7)	15 (88.3)	44 (70.9)	10 (12.5)
Neck pain	9 (13.6)	2 (12.5)	5 (11.4)	2 (11.1)
Duration of diabetes				
mean ± SD	9.0 ± 5.5	8.2 ± 4.3 ^a^	9.4 ± 5.8 ^b^	8.2 ± 5.8
range (years)	1–27	1–14	1–27	2–19
HbA1C (mmol/mol)				
mean ± SD	55.7 ± 7.7	55.6 ± 6.2 ^a^	55.8 ± 8.03 ^b^	53.6 ± 10.1 ^c^
range	43–75	44–64	43–75	43–70
Rheumatoid arthritis ^a^	8 (12.5)	3 (18.8)	4 ^a^ (10.3)	1 (14.3)
Osteoarthritis ^a^	33 (51.6)	9 (56.3)	22 ^a^ (52.4)	3 (33.3)
Stiff hand syndrome (%)	28 (42.4)	9 (56.3)	17 (38.6)	2 (28.6)
Neuropathic shoulder pain by DN4 (score ≥ 5)	2 (3.0)	0	1 (1.6)	1 (5.2)
Polyneuropathy				
Clinical	19 (28.8)	5 (29.4)	11 (19.2)	3 (15.7)
Subclinical	37 (56.1)	11 (64.7)	22 (38.5)	4 (21.1)

Values are presented as absolute numbers and percentages unless otherwise stated; SAPS: subacromial pain syndrome; GH: glenohumeral disorder; IQR: interquartile range; BMI: body mass index; SD: standard deviation; NRS: numerical rating scale; DN4: Douleur Neuropathique 4 questionnaire; ^a^ 2 are missing; ^b^ 4 are missing; ^c^ 1 is missing. ^#^ Statistical comparison between GH vs. SAPS: *p*-value < 0.0001, SAPS vs. Other disorder: *p*-value < 0.0001, GH vs. Other disorder: *p*-value 0.072.

**Table 2:** Ultrasound-diagnosed disorders in symptomatic and asymptomatic shoulders in patients with T2DM (*n* = 132).

Ultrasound-Diagnosed Disorders	All 66 Patients with 132 Shoulders					
	Symptomatic Shoulders (*n* = 93)			Asymptomatic Shoulders (*n* = 39)		
	*n*	%	95% CI	*n*	%	95% CI
Subacromial pain disorders	84	90.3	81.9–95.2	30	76.9	60.2–88.2
Subacromial bursitis	13	14.0	7.9–23.1	3	7.7	2.0–21.9
Rotator cuff disorder	84	90.3	81.9–95.2	30	76.9	60.2–88.2
LHBT disorder	10	10.7	5.5–19.3	3	7.6	2.01–21.9
Dynamic impingement	14	15.1	8.7–24.3	3	7.6	2.0–21.9
Glenohumeral disorders	8	8.6	4.1–16.7	0	0	0
Adhesive capsulitis	4	4.3	1.3–11.2	0	0	0
GH effusion only	4	4.3	1.3–11.2	0	0	0
Other disorders						
Acromioclavicular OA	55	59.1	48.4–69.1	17	43.5	28.1–60.2
No disorders	0	0	0	0	0	0

LHBT: Long head of biceps tendon; OA: osteoarthritis; GH: glenohumeral.

## Supplementary Materials

The following are available online at https://www.mdpi.com/2077-0383/9/12/4097/s1, Table S1: Criteria for ultrasound-diagnosed disorders groups, Table S2: Neurological feet examination procedures with diagnostic criteria, Table S3: Diagnostic criteria for stiff hand syndrome.
