# Supplementary material for: What Is the Diagnosis in Patients with Type 2 Diabetes Who Have a Painful Shoulder? Results from a Prospective Cross-Sectional Study
Source: J Clin Med. 2020 Dec 18;9(12):4097. doi: 10.3390/jcm9124097 (PMC7767012; doi:10.3390/jcm9124097)
Supplement: Supplementary file 1 [file jcm-09-04097-s001.pdf]

Supplementary data

Table S1: Criteria for ultrasound-diagnosed disorders groups

Ultrasound diagnosis	Diagnostic criteria
Subacromial disorders	
Subacromial-subdeltoid bursitis	Presence of any of the following criteria: bursal effusion >2mm, synovial proliferation, exhibition of Doppler signal, or a teardrop-shaped distal end of the bursa.
Calcifying tendinopathy	Presence of hyperechoic foci, with or without a well-defined acoustic shadow, or with a faint shadow in a rotator cuff tendon.
Tendinopathy	Focally or diffusely thickened rotator cuff tendon with a heterogeneous hypoechoic appearance.
Rotator cuff tears	
Partial-thickness tear	Presence of any of the following criteria: bursal or articular surface sided hypoechoic tendon discontinuity, or intratendinous mixed hyperechoic and hypoechoic region.
Full-thickness tear	Presence of any of the following criteria: focal tendon thinning, complete tendon non-visualization, focal tendon discontinuity with homogeneous echogenicity without focal thinning, or inversion of the superficial bursa contour and/or hyperechoic tissue.
Biceps pathology	
Tear	An anechoic cleft in the tendon (partial or complete) with fluid in the sheath or a non-visualisation.
Dislocation	Displacement over the lesser tuberosity and bicipital groove (subluxation), or completely out of the bicipital groove (dislocation).
Tendinopathy	Locally or diffusely thickened tendon with a heterogeneous hypoechoic appearance.
Tenosynovitis	Hypoechoic or anechoic thickened tissue with or without fluid within the tendon sheath, and which may exhibit Doppler signal.
Dynamic impingement	Bunging of the bursa or supraspinatus tendon on the coraco-acromial ligament, and/or anterior or lateral part of the acromion during dynamic assessment.
Glenohumeral disorders	
Adhesive capsulitis	Rotator interval proliferation with/without glenohumeral effusion without the presence of a rotator cuff tear.
Glenohumeral effusion	Presence of effusion in the glenohumeral joint.
Other disorders	
Acromioclavicular osteoarthritis	Presence of any of the following criteria: joint space narrowing, osteophytes, effusion, or para-articular cysts.

References are provided at the end of this file , references [1-3], but also included in the main manuscript.

Table S2: Neurological feet examination procedures with diagnostic criteria

Type of examination of the feet	Procedure	Diagnostic criteria
Inspection	Procedure: Look for clawed foot, hammertoes, and Charcot foot deformity, a bone deformity caused by neuropathy.	Presence of abnormality.
Muscle weakness test	Procedure: Test the flexion and extension strength of the small toes, and extension of the great toes.	Distal muscle weakness: ranging from no muscle contraction or any movement possible to less strength than expected.
Ankle reflex test	Tool: a reflex hammer.<br>Procedure: With the patient sitting, the examiner dorsiflexes the foot and gently strikes the Achilles tendon with the reflex hammer.<br>In the absence of reflex, the test will be repeated with reinforcement.	Abnormal reflex test: scored as zero (absent with reinforcement), one (present but decreased), two (normal), three (increased), or four (increased with clonus).
Touch sensation test	Tool: a Semmes-Weinstein monofilament.<br>Procedure: The monofilament is placed at four sites at the plantar side of the foot; big toe, first, third and fifth head of metatarsal bone. Repeat the test up to 3 times on an area when the patient does not indicate they feel the monofilament.	Abnormal sensation: when the patient feels the presence of the buckling monofilament at maximum 1 of the four mentioned sites even after the third time. .
Vibrating sensory test	Tool: a 128-Hz Rydel-Seiffer tuning fork.<br>Procedure: The tuning fork is placed at the dorsum of the interphalangeal joint of the big toes. The patient is asked to respond if he can no longer feel the vibration. At this time, the vibration is determined on the 9- point grading scale (0/8–8/8) of the tuning fork.	Below 4 points is considered as an abnormal vibration sense.

References are provided at the end of this file, references: [4-10], but also included in the main manuscript.

Table S3: Diagnostic criteria for stiff hand syndrome

Type of examination of the hand	Definition
Tabletop sign*	Inability to place the palms and fingers completely flat on a horizontal surface
Prayer sign*	Inability to fully extend the fingers

* We considered a stiff hand syndrome present if at least one sign was positive; References are provided at the end of this file, references: [11-13], but also included in the main manuscript.

Reference

1. Lee, J.C.; Sykes, C.; Saifuddin, A.; Connell, D. Adhesive capsulitis: sonographic changes in the rotator cuff interval with arthroscopic correlation. *Skeletal Radiol* **2005**, *34*, 522-527, doi:10.1007/s00256-005-0957-0.
2. Tandon, A.; Dewan, S.; Bhatt, S.; Jain, A.K.; Kumari, R. Sonography in diagnosis of adhesive capsulitis of the shoulder: a case-control study. *Journal of ultrasound* **2017**, *20*, 227-236, doi:10.1007/s40477-017-0262-5.
3. Ottenheijm, R.P.; Joore, M.A.; Walenkamp, G.H.; Weijers, R.E.; Winkens, B.; Cals, J.W.; de Bie, R.A.; Dinant, G.J. The Maastricht Ultrasound Shoulder pain trial (MUST): ultrasound imaging as a diagnostic triage tool to improve management of patients with non-chronic shoulder pain in primary care. *BMC Musculoskelet Disord* **2011**, *12*, 154, doi:10.1186/1471-2474-12-154.
4. England, J.D.; Gronseth, G.S.; Franklin, G.; Miller, R.G.; Asbury, A.K.; Carter, G.T.; Cohen, J.A.; Fisher, M.A.; Howard, J.F.; Kinsella, L.J., et al. Distal symmetric polyneuropathy: a definition for clinical research: report of the American Academy of Neurology, the American Association of Electrodiagnostic Medicine, and the American Academy of Physical Medicine and Rehabilitation. *Neurology* **2005**, *64*, 199-207, doi:10.1212/01.Wnl.0000149522.32823.Ea.
5. Alport, A.R.; Sander, H.W. Clinical approach to peripheral neuropathy: anatomic localization and diagnostic testing. *Continuum (Minneapolis, Minn.)* **2012**, *18*, 13-38, doi:10.1212/01.CON.0000411546.13207.b1.
6. Yang, Z.; Zhang, Y.; Chen, R.; Huang, Y.; Ji, L.; Sun, F.; Hong, T.; Zhan, S. Simple tests to screen for diabetic peripheral neuropathy. *Cochrane Database of Systematic Reviews* **2014**, 10.1002/14651858.CD010975, doi:10.1002/14651858.CD010975.
7. Feng, Y.; Schlösser, F.J.; Sumpio, B.E. The Semmes Weinstein monofilament examination as a screening tool for diabetic peripheral neuropathy. *Journal of vascular surgery* **2009**, *50*, 675-682, 682.e671, doi:10.1016/j.jvs.2009.05.017.
8. Smieja, M.; Hunt, D.L.; Edelman, D.; Etchells, E.; Cornuz, J.; Simel, D.L. Clinical examination for the detection of protective sensation in the feet of diabetic patients. International Cooperative Group for Clinical Examination Research. *Journal of general internal medicine* **1999**, *14*, 418-424, doi:10.1046/j.1525-1497.1999.05208.x.
9. Kästenbauer, T.; Sauseng, S.; Brath, H.; Abrahamian, H.; Irsigler, K. The value of the Rydel-Seiffer tuning fork as a predictor of diabetic polyneuropathy compared with a neurothesiometer. *Diabet Med* **2004**, *21*, 563-567, doi:10.1111/j.1464-5491.2004.01205.x.
10. Liniger, C.; Albeanu, A.; Bloise, D.; Assal, J.P. The tuning fork revisited. *Diabet Med* **1990**, *7*, 859-864, doi:10.1111/j.1464-5491.1990.tb01319.x.
11. Gokcen, N.; Cetinkaya Altuntas, S.; Coskun Benlidayi, I.; Sert, M.; Nazlican, E.; Sarpel, T. An overlooked rheumatologic manifestation of diabetes: diabetic cheiroarthropathy. *Clin Rheumatol* **2019**, *38*, 927-932, doi:10.1007/s10067-019-04454-z.
12. Hill, N.E.; Roscoe, D.; Stacey, M.J.; Chew, S. Cheiroarthropathy and tendinopathy in diabetes. *Diabet Med* **2019**, *36*, 939-947, doi:10.1111/dme.13955.
13. Fisher, L.; Kurtz, A.; Shipley, M. Association between cheiroarthropathy and frozen shoulder in patients with insulin-dependent diabetes mellitus. *Br J Rheumatol* **1986**, *25*, 141-146, doi:10.1093/rheumatology/25.2.141.
